# A nutritionally balanced menu for emergencies: beyond the COVID-19 pandemic

**DOI:** 10.3389/fnut.2026.1855250

**Published:** 2026-09-17

**Authors:** Nazuki Takahashi, Noriko Sudo, Gengaku Mashiro

**Affiliations:** 1 Faculty of Life Sciences, Ochanomizu University, Tokyo, Japan; 2 Natural Science Division, Faculty of Core Research, Ochanomizu University, Tokyo, Japan; 3 Corporate Affairs Group, Corporate Affairs Division, TOKYU CORPORATION, Tokyo, Japan

**Keywords:** boxed meal, COVID-19, hotel quarantine, infectious disease, nutrition, pandemic

## Abstract

**Introduction:**

During COVID-19 (a coronavirus infection) pandemic, hotels in Japan housed mildly ill patients due to the shortage of hospital beds. However, reports indicated the meals were of poor nutritional value. Hotel staff were inexperienced in preparing nutritionally balanced meals for convalescents. Hence, this study aimed to develop a model menu that considers both nutritional balance and employees’ responsibility. The case study was conducted of Hotel B in Tokyo, which operated as a hotel quarantine facility.

**Materials and methods:**

We evaluated the hotel’s menu, developed a model menu plan based on nutritional calculations, and conducted two semi-structured interviews with three decision-makers of Hotel B—the general manager and two chefs—regarding the feasibility of the model menu plan and modified model menu. We evaluated the nutritional value of bento box meals provided to patients and revealed excessive consumption of fried foods and meals that were insufficient in vitamin C, salty, and lacking in vegetables.

**Results:**

Hotel staff approved the feasibility of the modified model menu. The final suggested model menu consisted of dishes that were digestible and easy to prepare, no deep-fried main dish, and rich in fruits and vegetables. It also considered available resources, such as cost and personnel, and addressed concerns about hygiene and the aesthetics of the main dish. Identified restrictions were considered, including food storage and service. Additionally, logistics surrounding social distancing policies and the reassignment of infected patients and staff involved measures such as restricting elevator access.

**Conclusion:**

Swift and appropriate nutritional management of patients during a pandemic is important. Nutritionists should consider nutritional balance and other factors (e.g., safety and food hygiene, efficiency, and sensory appeal) when providing meals to recuperating patients.

## 1 Introduction

In December 2019, a coronavirus infection (hereafter COVID-19) was reported in Wuhan, China. By March 2020, the World Health Organization (WHO) declared the global spread of the infection a pandemic (1). In Japan, after the first positive case was confirmed on January 16, 2020, the rapidly spreading infection had impacted daily life and economic activities. Mobility restrictions led to the transition of schools and workplaces to online platforms and to the postponement of major events, such as the Tokyo Olympics. By the time the pandemic was downgraded to a Category 5 on May 8, 2023, close to 33.8 million people had tested positive. By then, approximately 75,000 deaths and 450,000 severe cases had been registered (2–4). The rapid increase in the number of infected people overwhelmed the public health system. Consequently, in April 2020, a system was implemented to provide care in hotels to mildly ill or asymptomatic patients who met specified criteria. Prefectures rented private accommodation facilities, such as hotels. It was also decided to recruit staff to provide healthcare and meals to patients recovering in isolation (5, 6).

Appropriate nutritional management is essential to prevent the progression of infections and to reduce the risk of mortality. Previous studies on the severity of the impact of nutritional status on viral infection have reported that malnutrition is associated with reduced resistance to infection and impaired immune responses (7–10). The WHO also published a document recommending adequate nutrition and avoiding excessive sugar, fat, and salt during the COVID-19 outbreak (11). Around that time, reports in Japan circulated about the inappropriateness of the meals provided to patients recuperating in hotel care facilities. Patients complained that the meals were “too greasy” and “lacking vegetables” (12–15). In a previous study on the meals provided to patients receiving care at hotels during the seventh wave of COVID-19 in Japan, the researchers found that more than half of the municipalities had received complaints about meals that were “greasy with a lot of fried food,” “rich,” “lacking fruits and vegetables,” and of “too large (portions),” which suggests that hotels were providing meals of unsuitable portions and content for medical treatment (16). Meanwhile, the hotel industry was facing its own challenges. Hotels had been experiencing significant labor shortages and high turnover rates even before the COVID-19 pandemic (17–20). With the onset of the disease, the situation was further aggravated by a reduction in staffing resulting from infections among family members and staff.

The use of hotel care facilities during infectious disease emergencies is stipulated in the Act on the Prevention of Infectious Diseases and Medical Care for Patients with Infectious Diseases (hereinafter referred to as the Infectious Diseases Act) and its enforcement regulations (21, 22). In the basic disaster management plan, hotel accommodations can also be used as shelters in the event of large-scale disasters, such as earthquakes or torrential rains (23, 24).

In Japan, meals provided in shelters during disasters were often inappropriate because they were deficient in protein, vitamins, and minerals (25–28). When malnutrition is combined with shock from the disaster and the stress of unfamiliar refugee life, there is a likely increase in the risk of contracting an infectious disease, worsening preexisting conditions and even causing disaster-related deaths (29–32). However, several limitations were evident in the operation of evacuation shelters (33, 34). These limitations were evident in hotel quarantines during the pandemic, emphasizing the importance of proper nutritional management in such settings during epidemics.

Researchers and the media reported that meals provided to patients recovering in hotels were nutritionally inadequate. These reports were supported by personal accounts of recovering patients and by previous reports evaluating the overall management of treatment at hotels (35–41); however, no studies have examined the specific contents and nutritional value of these meals. COVID-19 has been reclassified as a Category 5 pandemic in Japan. However, with the recent increase in global population, the concentration of urban dwellers, and the cross-border movement of people, a new pandemic is inevitable (42–45). Therefore, it is prudent to devise a plan for nutritional meals for convalescents in anticipation of such an eventuality.

The purpose of this study was to interview staff at a hotel that provided alternative accommodation for mildly ill patients and to develop a nutritionally appropriate bento box menu (hereafter referred to as the model menu). This model meal can be served during any emergency under various resource restrictions, such as during a pandemic or earthquake.

## 2 Materials and methods

### 2.1 Sampling

Our sampling strategy was purposive sampling. A certain hotel and people that could provide essential experience and information for this study were selected in a purposeful manner. Our purposive sampling is further classified into intensity sampling that selects information-rich cases that manifest the phenomenon of interest intensely (46). In this study, it is essential for us to select a hotel that could give us a data file of meals provided to patients receiving care at the hotel. Since it was not open information and one of corporate secrets, it was difficult to obtain cooperation from hotels selected by random sampling. In addition, collected menu data was going to be a basis for development of the model menu plan, we needed to select a hotel known as good care for patients. Due to these constraints, we employed a referral sampling and we were only able to get a single sample.

### 2.2 Target facility and participants

The research site was a hotel in city A in the Tokyo metropolitan area (hereafter referred to as Hotel B), which operated as a hotel care facility from July to the end of December 2021 (the fifth wave of COVID-19). During this period, the number of newly infected cases increased rapidly due to the emergence of a new variant (delta strain). The extremely high infectiousness and risk of serious illness placed further strain on the medical system; in response, the number of hotel rooms used as care facilities was expanded to more than 6,500 (47–49). Hotel B opened as the first hotel care facility in city A, accepting mild and asymptomatic patients in the city, with the concerted efforts of all employees, which has been highly evaluated within the group. This included its selection for the Grand Prix Environmental and Social Contribution Award among the more than 200 group companies to which Hotel B belongs (50). With Hotel B, we aimed to identify restrictions on the operation of residential sanatoriums and differences from standard operations, as well as exemplary practices that are worth adopting in other facilities in the future. Additionally, given the possibility of increased pandemics, model menus for use during the spread of infectious diseases have not yet been developed. Hence, this study aimed to practice them in advance at a single facility. Three staff members were invited to participate in the interview: general manager C, who was in charge at the time, and two chefs (D and E) who were involved in developing, preparing, and serving the menu. Although the number of interviewees was also limited to three, the manager and chefs were the decision makers on management and operation, respectively.

### 2.3 Procedure

The study was conducted in the following steps:

#### 2.3.1 Dietary assessment of the meals provided by hotel B

First, we obtained a data file of 1 week’s worth of bento box meals provided by Hotel B to patients receiving care at the hotel at that time (hereinafter referred to as boxed meal at that time) and conducted dietary assessments. The file listed the menu name, the ingredients and their amounts, the per capita food cost, and 28 allergies for breakfast, lunch, dinner, and snacks. Smart nutrition calculation software (Ver. 9.2) (Ishiyaku Publishers, Inc., Tokyo, 2025) with the Standard Tables of Food Composition in Japan, 2020 (8th revision; (51)) was used for the nutrition calculations. For dishes, the nutritional information of their ingredients and quantities was included in the file, it was used for nutrition calculations. For dishes for which such information was not listed, the values listed in the Standard Tables of Food Composition in Japan as “prepared foods for distribution” were used. For the values not listed, the ingredients and quantities in recipes, such as the “Sonomanma Bento Card,” were used as a reference (52, 53). The indices used to evaluate the nutritional calculation results are shown in Table 1. The Nutritional Reference Values (hereafter referred to as RVs) for Feeding at Evacuation Shelters, which are the nutrition standards for emergencies, were first published in 2011 by the Japanese Ministry of Health, Labour and Welfare (54). They were revised by Sudo et al. (55) and Sato et al. (56) who worked in a research team funded by the Ministry in 2020–2021. Revised values were also updated in accordance with new values in the latest Dietary Reference Intakes for Japanese 2025 (57) for energy, protein, vitamin B_1_, vitamin B_2_ and vitamin C, and the national health promotion plan, “Health Japan 21 (the third term)” for FY2024-2033 (58) for the salt equivalent (hereafter salt). These final values were used for this study (Table 1).

**Table 1:** Nutritional reference values (RV) used in this study.

Nutrients	RV
Energy^*1^	2,000 kcal or more
Protein^*1^	55 g or more
Vitamin B_1_^*1^	1.00 mg or more
Vitamin B_2_^*1^	0.60 mg or more
Vitamin C^*1^	80 mg or more
Salt equivalent^*2^	less than 7.0 g

The Nutritional Reference Values for Feeding at Evacuation Shelters, which are the nutrition standards for emergencies, were first published in 2011 by the Japanese Ministry of Health, Labour and Welfare. They were revised by Sudo et al. and Sato et al. who worked in a research team funded by the Ministry in 2020–2021. Revised values were also updated in accordance with value changes in the Dietary Reference Intakes for Japanese 2025^*1^ and the national health promotion plan, “Health Japan 21 (the third term)” ^*2^ for FY2024-2033.

Although RVs are also presented by sex and age group as a reference, the most widely used is the values for ages one and up weighted by whole Japanese population. This is because in mass feeding, individuals’ sex and age were hardly considered in the past disaster scenes (55). In this study, we also used the values for person 1 year old and over since it was also an emergency situation.

In addition to nutritional calculations, the frequency of fried food and vegetable servings was tabulated. Fried is defined as “a cooking method in which food is heated at high temperatures using a large amount of cooking oil” (59). Vegetables, following multiple references, were classified into raw/salad, boiled/steamed, with dressing, pickled, boiled, simmered, soaked, fried, grilled, stir-fried, and soup (including those served in liquid form, such as vegetable juice), (53, 60–62).

#### 2.3.2 Development of a model menu plan

In response to the issues identified in the previous section (2.3.1), a model menu plan was developed that meets the RVs.

#### 2.3.3 Two interviews

Semi-structured interviews of approximately 2 h each were conducted on August 21 and October 16, 2025, via Zoom. Questions in the two interviews are shown in Table 2. At the interviews, the first author, the menu developer, read aloud the interview script that was sent to the participants in advance. She was an undergraduate student of registered dietitian course. The interviewees, especially two chefs, acted like mentors who gave her professional advice on her menu. When they seemed reserved to provide candid feedback, the second author, who was her academic supervisor, facilitated and clarified their true opinions. After the first interview, the model menu plan was revised, and the modified model menu was cooked and photographed for use as interview material in the second interview. The interviews were recorded with participants’ consent using Zoom’s recording function, and the recordings were then transcribed by a professional transcription service. After the participants reviewed the transcribed interviews, their answers to the questions were summarized and reflected in modifications conducted twice, resulting in the final suggested model menu (Figure 1).

**Table 2:** Questions and answers in two interviews.

Question	Answerer	Answer
1st interview (August 21, 2025)		
Regarding Hotel B’s operation, restrictions, and consideration		
In the preparation and serving of the box meals, what aspects were different from normal hotel operations, were challenging, or required special consideration?	Chef E	I think this is a fundamental issue, but since it’s a homemade bento, hotels also provide bento boxes, but usually, we prepare and serve them to guests so they can eat them right away. However, in the case of takeout bento like this one, there were often very few restrictions. First, the core temperature must be set to the specified temperature, which led to many dishes being fried or sautéed in oil, as you mentioned. I tried to avoid adding as much water as possible, so I gave up on soup as well. There were many restrictions far removed from regular operations, and for us, it was the biggest mental burden, I think.
There were various conditions and restrictions imposed by the local government. What difficulties did you face in providing meals under those conditions?	Manager C	Upon offering the facility, the Tokyo Metropolitan Government inspected the facility, and it was decided to utilize not only the guest rooms, but also the banquet hall on the third floor and its associated kitchen. The banquet room on the third floor was used to serve meals and as a staff station. The corridors and elevators used by patients were not available to our partner companies or us. All corridors, elevators, stairs, etc. were separated from those receiving medical treatment, and measures were taken to zone them. Therefore, ingredients and equipment could no longer be brought up and down using the elevators that we normally use, and, instead, we had to use the stairs. And the storage space was half that of a normal food storage area.
	Manager C	For breakfast at restaurants, we usually serve about 50 to 60 meals, but on busy days or when we are taking orders for groups, we serve between 100 and up to 150 to 160 meals. However, since this is a buffet service, it is not a boxed meal as we did this time. We also served lunch and dinner at restaurants and accepted banquet orders, but serving about 120 to 150 meals in the form of bento boxes for breakfast, lunch, and dinner at a lodging facility for two to three consecutive weeks was quite a considerable amount for us.
What were the primary considerations in menu preparation?	Chef E	The primary goal is that the health of our residents must not be compromised by the meals we provide. Safety and security—this was our top priority. And among them, as chefs, we always thought about making the presentation look beautiful and as delicious as possible.
Regarding the model menu plan for Day 1		
How much time is expected to be required to prepare, distribute, and serve the model menu proposed by the authors?	Chef E	It’s quite difficult to answer this question, so I’d like to talk about the environment we were in at the time and our staff. At that time, the designated service hours for breakfast, lunch, and dinner were 7:30 a.m., 11:30 p.m., and 5:30 p.m., respectively. They said to keep a maximum of 150 meals in mind, with punctuality in mind. For staffing, the confirmed number of staff was: one night shift and one after night shift, with two starting at 6 a.m., one at 8 a.m., one at 10:30, and two or three at 11 a.m., totaling 6 to 7 people per day, each working about 8 h, totaling nine people in the shift at the time.
	Chef E	For the breakfast, we cooked at night, let them cool, and finished as a bento overnight. They were stored in the refrigerator and served in the morning. We cooked them once, then quickly cooled them and served them chilled. Then, we hand it to them cold, and when patients come to pick up, the Tokyo Metropolitan Government has prepared about eight microwaves, so patients warmed them up there before taking them to their room.
We would like to provide one carton of grapefruit juice and milk for each person with this menu. Would this be difficult to achieve given purchasing costs?	Manager C, Chefs D and E	This depends on the budget now, but as long as you have a solid budget, the content is not that difficult.
Are there any difficulties in terms of the number of menus or the preparation process for the other menus?	Chef E	See Tables 2, 6 for Spanish omelet (2nd row) and salted rice ball (6th row).
Regarding the model menu plan for Day 2		
A lot of fruits are to be used. Fruits are expensive and spoil quickly. Is it feasible to provide these, given their purchasing cost and shelf life?	Chef D	Bananas are served as is, and when patients come to pick them up, they put them in a bag and take them to their room. If you cut it in half, it may discolor or bacteria may get in, so if you can take it to your room as a whole with a regular bento, this is possible.
Regarding the model menu plan for Day 3		
Rice with green soy beans is intended to be prepared and served as mixed rice. Is this method feasible for serving a large number of people in a timely manner?	Chef D	See Tables 2, 6 for rice with green soy beans (3rd row).
Is there any other difficult menu?	Manager C	See Tables 2, 6 for meat and potato stew (11th row).
We would like to provide two snacks and are considering two options: (1) between breakfast and lunch and between lunch and dinner, or (2) between lunch and dinner and after dinner. Is this feasible from the perspectives of the timing of serving and the budget?	Chef E	In between each meal service time, we had to disinfect and clean up the serving site. Additionally, at the boxed meal serving location, procedures for admission and discharge of residents were also conducted. There was no time to provide snacks between meals, which I think was tough.
	Manager C	The admission and discharge times were fixed daily, roughly in the morning and afternoon. The area where the patients entered and exited had to be cleaned with protective clothing on, so it was difficult at that time to set the time to give three meals plus a snack. Therefore, we had to serve a small dessert with the boxed meal.
Regarding the model menu plan for Day 4		
What were the difficulties in implementation in terms of manpower, time and effort required for cooking, costs, and operations?	Chef E	The first thing we considered was the selection of containers, since there are differences in volume between breakfast, lunch, and dinner. In retrospect, it seems to have contributed to work efficiency and accident prevention, so we think it would be more practical to consider standardizing the containers. To buy one type of container, I think the minimum lot was 800 units. Looking at the containers in the model menu plan, I was concerned that the containers for breakfast, lunch, and dinner might get mixed up, and it would be easy for minor accidents to occur.
	Manager C	We wanted to order a variety of containers, but ordering so many different types would have limited our storage options, and we did not have a storage space or anywhere to store them. It’s not too good to leave them out in the open. As that happened, we kept the lunch box with four or five compartments and one separate container, a round plastic case for salad.
	Chef E	See Tables 2, 6 for rice with salmon (3rd row), pork shabu-shabu somen noodle (4th row), and cabbage wrapped in pork (7th row).
Regarding the model menu plan for Day 6		
What were the difficulties in implementation in terms of manpower, time and effort required for cooking, costs, and operations?	Chef D	See Tables 2, 6 for mackerel simmered in tomato sauce (8th row).
Regarding the model menu plan for Day 7		
What was the number one reason for stopping the provision of soup during the COVID-19 pandemic? In addition, from the perspective of preventing the spread of infection, what are some other ideas about how to provide it? (see “corn soup” in Tables 2, 6)	Manager C	One reason we could not serve soup was that leftovers after eating end up being flushed in the bathtub, washbasin, or toilet on their own, which we have felt as a risk. Considering such risks, we deliberately did not serve any soup dishes.
	Manager C	Even if things like garbage disposal are resolved, the cold soup is still heated by the patient in the microwave. The method of customers taking soup from the zundo hot pot served at standing places in a ladle is also a hygiene concern.
	Chef E	Bringing it with a lid carries a high risk of liquid leakage and is tough, and the style of a regular standing restaurant is problematic.
What were the difficulties in implementation in terms of manpower, time and effort required for cooking, costs, and operations?	Manager C, Chef E	See Tables 2, 6 for spinach and egg drop soup (2nd row), rice with bamboo shoots (3rd row), stewed pumpkin with minced meat (9th row), and apple (10th row).
2nd interview (October 16, 2025)		
Regarding the modified model menu for Day 1		
What are your concerns regarding the appearance and portions for each menu?	Chef E	As we discussed last time (the first interview), our menu at the time was “conventional in appearance and volume,” while your menu seemed to “be healthy, nutritious, and good for the body” and “have a lot of vegetables.” Besides, I am concerned that there are hardly any heavy dishes. In creating our menu, we thought it was important to think of what people would base their bento box purchase on. Like their mood of the day, what they want to eat, what they ate before, and what they will eat later. Since these meals were for recuperating patients who could not choose the meal, we first considered making the main dish impactful, and then maintained a balance of meat, fish, and vegetables in the side dishes, so that we could cater to different tastes.
	Chef D	I think it’s nice to have plenty of vegetables for breakfast, carrot is always roughly chopped and boiled. For example, how about making carrots into purees or trying different variations? (see “vegetables-carrots” in Tables 2, 6)
Is it possible to serve “Inari Sushi” for lunch using ready-made or frozen foods?	Chef E	Yes, it is. I have a suggestion. I have never seen pickles on the menu, so I think it would be okay, to add pickled ginger here (see “sushi wrapped in fried tofu” in Tables 2, 6).
Regarding the modified model menu for Day 2		
Which type of container is preferable for lunch? Is it feasible to provide containers of an unconventional shape?	Chef D	The conventional container is fine. In addition, it is preferable to provide individually wrapped servings (see “sandwich” in Tables 2, 6).
Do you have any additional opinions or suggestions?	Chefs D and E	See Tables 2, 6 for boiled egg (13th row).
Regarding the modified model menu for Day 3		
Is it permissible to use and serve chilled tofu for dinner, even though it is uncooked tofu?	Chef E	See Tables 2, 6 for chilled tofu (14th row).
Regarding the modified model menu for Day 4		
Is it possible to prepare udon for lunch using this method?	Manager C, Chefs D and E	See Tables 2, 6 for mackerel udon noodles (4th row).
Regarding the modified model menu for Day 5		
Do you have any additional opinions or suggestions?	Manager C, Chef E	See Table 2 for udon noodle salad (17th row).

**Figure 1:**
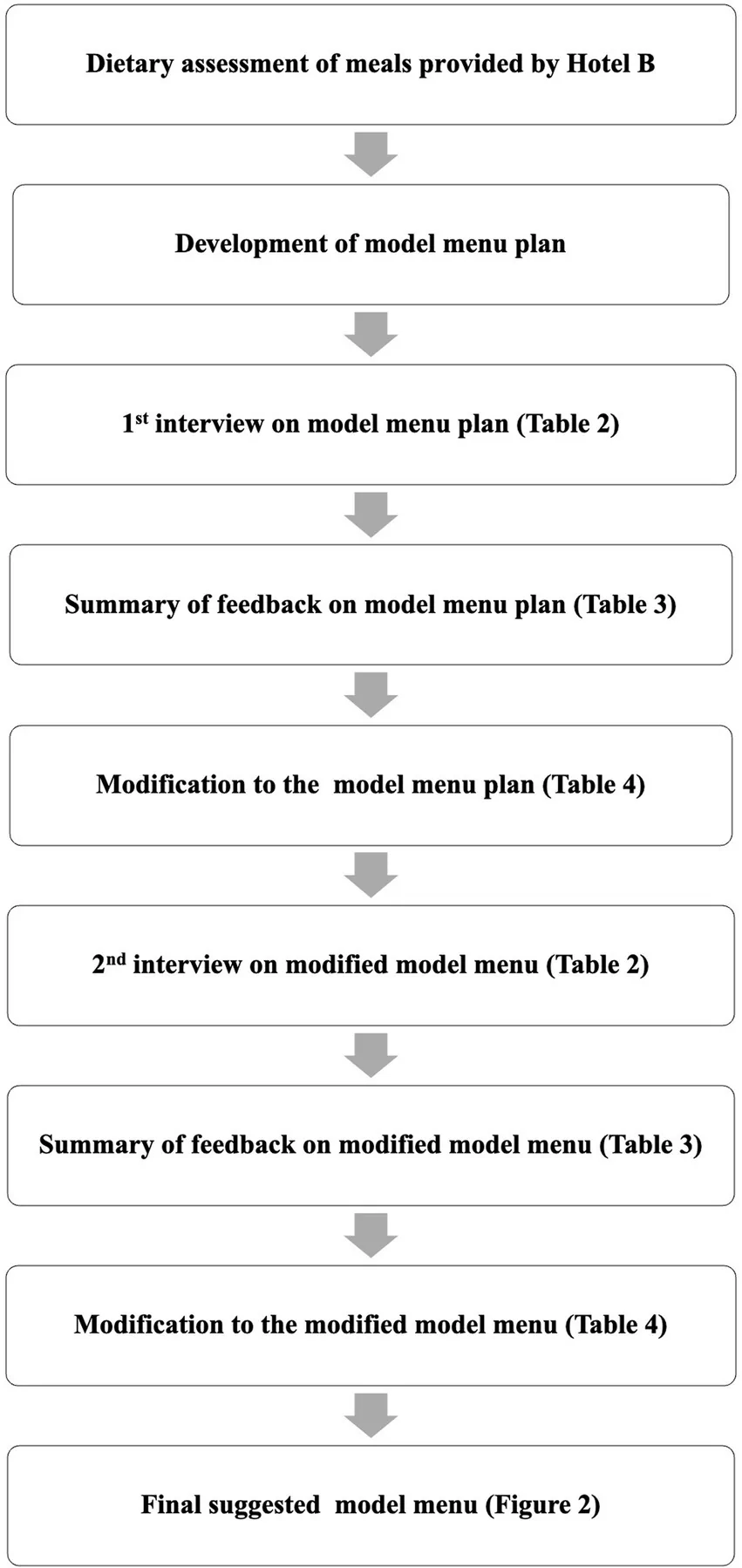
Process to make final suggested model menu.

#### 2.3.4 Evaluation of the final suggested model menu in comparison with the “smart meal pattern”

In Japan, the recent increase in demand for ready-to-eat foods (63) prompted the Healthy Food Environment Consortium to announce the “Smart Meal Standard” (64, 65). We compared energy and nutrient contents in the final suggested model menu with the “Smart Meal Pattern” shown in Table 3. We described which meals failed to meet the established criteria and presented the percentage of the meals.

**Table 3:** Comparison of the final suggested model menu with Smart Meal criteria.

Smart Meal criteria^*1^	Moderate^*2^	Substantial^*3^	Meals that did NOT meet either Moderate or Substantial standard	%^*4^
Energy	450–650 kcal	620–850 kcal	None	0%
Meal combination	(1) Staple + main dish + side dish or (2) Staple + side dish (main dish, side dish)		None	0%
Protein–fat–carbohydrate balance (energy ratio)	Protein 13–20% Fat 20–30% Carbohydrates 50–65%		Day1L, Day1D, Day2B, Day3L, Day4B, Day5B, Day5D, Day6B, Day6L, Day6N, Day7B, Day7L^*5^	57%
Weight of vegetables, mushroom, seaweed, and potato	140 g or more		Day1B, Day2B, Day3L, Day3D, Day5B, Day5D, Day6B, Day6L, Day7B, Day7L, Day7D	52%
Salt equivalent	Less than 3.0 g	Less than 3.5 g	None	0%
Milk, dairy products and fruits	No criteria but should be consumed as appropriate.		None	0%
Foods and ingredients intended to serve specific health purpose	Do not use. (However, the use of low-sodium soy sauce, etc. is permitted.)		None	0%

^*1^ Source: Issued by the Consortium for Healthy Meal and Food Environment General Incorporated Association, 2015. Based on the ‘Dietary Guidelines for Preventing Lifestyle Related Diseases and Other Health Promotion” (September 2015) by the Ministry of Health, Labour and Welfare, and prepared with reference to other dietary intake standards and research results (evidence) related to healthy eating. An example of a case in which one third of the estimated daily energy requirement listed in the Dietary Reference Intakes for Japanese (2020 edition) is considered adequate. ^*2^ Moderate: For women in general who want to eat ‘properly’ with good nutritional balance in mind. ^*3^ Substantial: For men and women who want to eat a ‘substantial’ diet with a good nutritional balance in mind. ^*4^ Proportion of 21 meals (3 meal per day over 7 days). ^*5^ Alphabets (B, L, and D) following day number show breakfast, lunch, and dinner, respectively. For example, “Day3B” stands for “Breakfast on Day 3”.

### 2.4 Ethical considerations

The Ethical Review Board of Humanities and Social Sciences Research at Ochanomizu University approved the study (Approval date: July 10, 2025, Approval number: 2025–60). Participants were sent a research cooperation request form outlining the survey’s objective and the publication method. Participants provided signed informed consent. In appreciation of their time spent in interviews, we donated 50,000 yen to the company to which C now belongs and 80,000 yen to the hotel to which D and E belong.

## 3 Results

### 3.1 Dietary assessment of boxed meals served at hotel B during the fifth wave

Nutritional calculations were made for 7 days of boxed meals shown in Table 4. Dietary assessment based on the RVs is shown in Table 5.

**Table 4:** Menu of boxed meals served at Hotel B during the fifth wave.

Meal	Day 1	Day 2	Day 3	Day 4	Day 5	Day 6	Day 7
Breakfast	Croissant Bread roll Scrambled eggs Sausages Hams French fries Okra Fruits	Sandwich Meatballs Hash brown potatoes Pickles Broccoli Cherry tomatoes Yogurt	Croissant Bread roll Boiled egg Bacons Fried Takoyaki Hash brown potatoes Broccoli Fruits	Sandwich Fried chickens French fries Pickles Broccoli Cherry tomatoes Yogurt	Croissant Bread roll Scrambled eggs Sausages Bacons French fries Okra Yogurt	Sandwich Fried chickens French fries Pickles Broccoli Cherry tomatoes Fruits	Croissant Bread roll Boiled eggs Bacons Hams Hash brown potatoes Yogurt
Lunch	Rice Ginger pork stir-fry Grilled mackerel Macaroni salad Pickles Spicy konjac Cauliflower Salad	Rice Fish with sweet and sour sauce Pork cutlet Fish cake Simmered radish Potato salad Pickles Vegetables Salad	Rice Fried chicken with grated radish Fried fishes Yakisoba Pickles Broccoli Cherry tomatoes Salad	Rice Sautéed fish in tomato sauce sautéed onions Meatballs Pickles Potato salad Boiled carrots Cauliflower Salad	Rice Chicken Nanban Grilled salmon Tomato ketchup Pasta Pickles Kinpira Salad	Rice Grilled fish with Miso sauce Mench cutlet Fish cake Stir-fried hijiki Macaroni salad Pickles Broccoli Salad	Rice Fried squid Fried fishes Fried shrimp Chicken cutlet Japanese-style dashi pasta Shumai Pumpkin Pickles Salad
Dinner	Rice Chinese-style fried chicken Braised pork belly Thick omelet Shumai Spring rolls Pickles Broccoli	Rice Beef Yakiniku Deep fried tofu Peas Thick omelets Teriyaki Chicken Fried squid Pickles Sweet potatoes	Rice Grilled salmon with Miso sauce Taro dumplings with savory sauce Mench cutlet Meatballs Thick omelets Broccoli	Rice Pork cutlet Teriyaki Chicken Grilled salmon Thick omelets Pickles Simmered radish Potatoes	Rice Teriyaki yellowtail Fried chicken Shumai Simmered fried tofu pouches Thick omelets Pickles Sweet potatoes	Rice Hamburger steak with melted cheese inside Fried fishes Meatballs Thick omelets Fried tofu Pickles Shishito Pumpkin	Brown rice Fish with sweet and sour sauce Cabbage rolls Pork cutlet Thick omelets Kinpira Pickles Carrots Broccoli

**Table 5:** Energy and nutrient contents of boxed meals served at Hotel B during the fifth wave.

Day	Energy (kcal)	Protein (g)	Vitamin B_1_ (mg)	Vitamin B_2_ (mg)	Vitamin C (mg)	Salt equivalent (g)
1 2 3 4 5 6 7	2,635 2,172 2,468 2,418 2,815 2,450 2,551	91 57 87 85 83 90 80	1.25 0.91 1.43 1.10 0.94 1.69 1.18	1.05 0.75 1.04 1.35 0.97 1.40 1.29	84 120 69 62 67 70 73	12.6 16.9 10.1 11.1 13.1 11.2 11.8
Mean (min, max)	2,501 ± 184 (2,172, 2,815)	82 ± 11 (57, 91)	1.21 ± 0.25 (0.91, 1.69)	1.12 ± 0.22 (0.75, 1.40)	78 ± 18 (62, 120)	12.4 ± 2.1 (10.1, 16.9)
Coefficient of Variation	0.07	0.13	0.21	0.19	0.23	0.17
Reference Values	2,000 kcal or more	55 g or more	1.00 mg or more	0.60 mg or more	80 mg or more	Less than 7.0 g

#### 3.1.1 Frequent service of fried foods

Of the 21 breakfast, lunch, and dinner meals prepared over 7 days, 19 (90.5%) were fried. French fries and hash browns were served every other day for breakfast, while deep-fried crumbed meat patties and other deep-fried crumbed dishes were served for lunch and dinner. On some days, four kinds of fried foods were served in one meal under the menu name of “assorted fried food.” The fat energy ratio on those days was 36%.

#### 3.1.2 Inadequate provision of vegetables

Although vegetables were served every day, the average amount served was 234 g over the 7-day period. Additionally, the daily amounts varied widely, from 148 g to 322 g.

In terms of cooking method, raw/salad was the most common (29.8%), followed by fried (20.5%) and boiled/steamed (19.3%). Raw/salad, boiled/steamed, pickled, and fried vegetables were served daily. Simmered vegetables were served on 6 out of 7 days and vegetables with dressing on 4 out of 7 days, while stir-fried vegetables were served only once in the form of sauteed onions. Pickled vegetables were prepared Japanese style, such as “tsubozuke” and “shibazuke,” and in Western style, such as “pickles.”

#### 3.1.3 Insufficient supply of vitamin C

Vitamin C contents below RV were observed on 5 out of 7 days. Broccoli was served at all three meals on the only day during the 7-day period when a vitamin C supply of 120 g was observed.

#### 3.1.4 Excessive salt

The RV of less than 7.0 g was exceeded on all 7 days. The salt equivalent was 10.0 g (143% of the RV) on the lowest day, with 16.9 g (241%) on the highest day. On some days, nearly 2.5 times the RV of salt was used.

### 3.2 Designing a model menu plan

Based on the above results, a model menu plan was developed using the dishes actually served at the hotel. There was a focus on “increasing the intake of vegetables and vitamins” and “using cooking forms and methods that are easy to eat even in poor health.”

### 3.3 Interview results

#### 3.3.1 Feasibility in limited resources

Feedbacks are shown below when asked if our model menu plan (1st interview) and modified model menu (2nd interview) was feasible from the perspective of each of the following resources.

##### 3.3.1.1 Personnel

At that time, Hotel B operated as a care facility, providing up to 150 meals per day, with six to seven staff members, each working 8 h per day, and a total of nine kitchen staff (Table 2). The work assignments were arranged according to the time of day, as follows: 6:00 a.m. workers check the number of breakfast boxes and serve them, and then cook lunch; 11:30 a.m. workers serve lunch, then cook dinner and breakfast for the next day; and night shift workers check the number of breakfast boxes. The work was coordinated with three to four workers for breakfast, two for lunch, and 4–5 for dinner. Furthermore, the responses indicated that “since lunch had to be served by the designated time with two staff members coming in at 6:00 a.m. and two staff members coming in at 7:00 a.m., it was difficult under the circumstances at that time to serve a number of different kinds of labor-intensive dishes for lunch.”

Regarding the timing of snacks, the interviewees provided the following responses, which referred to the facility management situation at the time:

Chef D: “In between each meal service time, we had to disinfect and clean up the serving site. Additionally, at the boxed meal serving location, procedures for admission and discharge of residents were also conducted. There was no time to provide snacks between meals, which I think was tough.”

Manager C: “The area where the patients entered and exited had to be cleaned with protective clothing on, so it was difficult at that time to set the time to give three meals plus a snack. Therefore, we served a small dessert with the boxed meal.”

Given the number of disinfecting procedures performed between meals and the number of staff required to perform them, providing a separate snack was difficult. Hence, it was preferable to serve it as a dessert with meals.

##### 3.3.1.2 Budget

The average daily cost of boxed meals at Hotel B was 1,242.7 yen, or an average of 414.2 yen per meal. On the other hand, the average daily cost of preparing the model menu plan was 2,022.8 yen, including containers and beverages, with an average cost per meal of 640.4 yen. After presenting this budget, we asked about the financial feasibility of the model menu plan. Interviewees responded that “it was feasible under the same conditions as the COVID-19 disaster because it was within the budget. The Tokyo Metropolitan Government requested that the standard meal cost be “1,000 yen per meal, including the cost of containers and beverages.”

##### 3.3.1.3 Movement line and equipment

When asked about the feasibility of the model menu plan in terms of movement line and equipment, the following conditions were described. The participants responded that “it was feasible under the circumstances and conditions of the time.”

Manager C: “Upon offering the facility, the Tokyo Metropolitan Government inspected the facility, and it was decided to utilize not only the guest rooms, but also the banquet hall on the third floor and its associated kitchen. The banquet room on the third floor was used to serve meals and as a staff station. The corridors and elevators used by patients were not available to our partner companies or us. All corridors, elevators, stairs, etc. were separated from those receiving medical treatment, and measures were taken to zone them. Therefore, ingredients and equipment could no longer be brought up and down using the elevators that we normally use, and, instead, we had to use the stairs. And the storage space was half that of a normal food storage area” (Table 2).

The interviewees shared that, due to the aforementioned zoning, “we could not use the rice robot (industrial rice cooker) that we usually use, and we had to use three 1.8 L rice cookers to serve rice.”

##### 3.3.1.4 Time

Chef E stated that “meals were served punctually with breakfast at 7:30 a.m., lunch at 11:30 a.m., and dinner at 5:30 p.m.” They also stated that “breakfast was cooked and cooled the day before and then packed as a boxed meal.”

When asked if it would be possible to provide the model menu plan within the period, the interviewees responded that “it would be possible if there were enough personnel allocated for the content.”

#### 3.3.2 Feedback on appearance and volume

Chef E commented that while the hotel’s boxed meals were “conventional in appearance and volume,” the model menu plan and modified model menu seemed to “be healthy, nutritious, and good for the body,” and “have a lot of vegetables” (Table 2). In particular, he stated the following: “I am concerned that there are hardly any heavy dishes.”

Chef E: “In creating our menu, we thought it was important to think of what people would base their bento box purchase on. Like their mood of the day, what they want to eat, what they ate before, and what they will eat later. Since these meals were for recuperating patients who could not choose the meal, we first considered making the main dish impactful, and then maintained a balance of meat, fish, and vegetables in the side dishes, so that we could cater to different tastes.”

#### 3.3.3 Feedback for improving each menu

The interviewees gave us feedback for menu improvement mainly from three perspectives: work efficiency, safety and security, and deliciousness (Table 6).

**Table 6:** Feedback from Hotel B staff on the model menu plan (1st interview on Aug 24, 2025) and modified model menu (2nd interview on Oct 16, 2025).

Menu name	When opinion collected	Opinion details
Spinach and egg drop soup	1st interview	It is unsure whether it will be properly heated in the middle, which is undesirable from the viewpoint of putting it in the bento box. Egg soup is not hygienic because of the juices that are released.
Spanish omelet	1st interview	
Rice with green soy beans	1st interview	Rice cooked with mixed ingredients carries the risk of uneven cooking. It is preferable to mix the ingredients into the cooked rice or add it on top.
Rice with salmon	1st interview	
Rice with bamboo shoots	1st interview	
Pork shabu-shabu somen noodles	1st interview	If leftover noodle soup is poured down the drain in the guestroom, it may damage the drain pipe. When boiling somen noodles, the moisture released from them can be a source of bacterial growth.
Mackerel udon noodles	2nd interview	
Corn soup	1st interview	The following points were raised as concerns when serving soup. (1) If patients serve themselves from a pot, there is a risk of spreading infection by sharing utensils, such as the ladle. (2) Easy to spill during transportation and poor work efficiency. (3) Facility concerns about disposing of leftover soup and ingredients in the guestroom toilets and sinks.
Salted rice ball	1st interview	Because the food is hand-held, there is a risk of food poisoning bacteria such as *Staphylococcus aureus* spreading.
Meat-stuffed cabbage	1st interview	It is more efficient to eliminate the wrapping process. For example, using the mille-feuille format would not change the taste, but would simplify the process.
Mackerel simmered in tomato sauce	1st interview	Mackerel is a fish that tends to leave a strong odor. Instead of cooking it as simmered in tomato sauce. It is preferable to pour the tomato sauce over the pre-baked fish.
Stewed pumpkin with minced meat	1st interview	In the case of stewed minced meat water is released in the bento box. It is preferable and more efficient to use stewed bean paste and pour the bean paste over the pumpkin.
Apple	1st interview	Fruits that are prone to discoloration were not used very often because the discoloration gives the impression of deteriorated taste and appearance.
Meat and potato stew	1st interview	If cooked in bulk, it would have been difficult to distribute the ingredients evenly with the number of staff available at the time. By cooking and serving each ingredient separately, the ingredients can be evenly distributed to everyone.
Sandwich	2nd interview	It is preferable to provide individually wrapped servings because bread loses both texture and flavor when it dries out, and dry bread also absorbs moisture from other ingredients, which is also a concern from a hygienic standpoint.
Boiled egg	2nd interview	It is more colorful when cut in half, but there is a risk of fungal growth on the cut surface. It is preferable to serve uncracked and unshelled.
Chilled tofu	2nd interview	When using tofu, it is best to cook it first before serving for hygienic reasons.
Sushi wrapped in fried tofu	2nd interview	I have never seen pickles on the menu, so I think it would be okay to add pickled ginger here.
Vegetables-Carrots (boiled)	2nd interview	It seems to be hard to eat because there are so many just-boiled vegetables. -Try serving them as carrot puree, grated carrot, etc.
Udon noodle salad	2nd interview	The container is full of ingredients, and then adding the noodles and mixing would be hard to eat. It would be good to have a separate container for eating.

##### 3.3.3.1 Work efficiency

The interviewees stated that, for cooking large quantities, “it would be more efficient to prepare the ingredients and sauce separately for thick sauce dishes, then pour the sauce over the ingredients when serving” and that “if the same ingredients and seasoning are used, it is preferable to choose a cooking method that requires less time.” Additionally, for simmered dishes, such as meat and potato stew, “after cooking them all together, it takes time to pick out each ingredient from the pot and arrange them evenly.” As an alternative, it was suggested to “cook, season, and arrange each ingredient separately.”

##### 3.3.3.2 Safety and security

Safety and security were the most frequently discussed topics among participants during the interviews. Comments regarding most of the menus included “concern about uneven heating,” “risk of growth of food poisoning bacteria and other germs associated with manual handling,” “food should be heated before serving,” “minimize moisture and juice as much as possible,” and “concern about serving soup.”

##### 3.3.3.3 Deliciousness

Opinions regarding deliciousness were primarily obtained in the second interview. Comments included “a recommendation to vary menu items rather than having the same items repeatedly” and “concerns about changes in texture and deterioration over time.”

### 3.4 Modifications to the menus and creation of final suggested model menu

Based on the interview participants’ opinions, modifications were made to the menus (Table 7). The final suggested model menu is shown in Table 8, and two sample photographs are presented in Figure 2. The boxed meals served by Hotel B at the time and the final suggested model menu differed in the following two respects:

**Table 7:** Modifications to the menus through two interviews with Hotel B staff.

Original menu	Modified menu	When modification made^*1^	Justification for modification
Spinach and egg drop soup Spanish omelet	Sauteed eggs and spinach	After 1st interview	By using stir-fry as the cooking method, it is possible to reduce the risk of uneven heating.
Rice cooked with green soy beans Rice cooked with salmon Rice cooked with bamboo shoots	Boiled rice mixed with edamame Boiled rice mixed with salmon Boiled rice mixed with bamboo shoots	After 1st interview	The use of cooking rice and then adding ingredients instead of cooking rice and ingredients together eliminates concerns about unevenness in cooking.
Pork shabu-shabu somen noodles Mackerel udon noodles	Pork salad with udon noodles Mackerel salad with udon noodles	After 1st interview After 2nd interview	Instead of pouring the soup over the udon noodles, the udon noodles were made into salad udon and mixed with the seasoning liquid to reduce the amount of liquid remaining in the udon. A container for holding the ingredients and a container for eating were prepared to make it easier to eat.
Corn soup	(Not served)	After 1st interview	Due to concerns over how to dispose of leftover liquid, corn, and croutons, the product stopped being offered.
Salted rice ball	Sushi wrapped in fried tofu	After 1st interview	Due to the risk of food poisoning associated with hand-formed rice balls, the product was changed to ‘inari-zushi,’ which is rice stuffed into fried bean curd.
Meat-stuffed cabbage	Pork and cabbage mille-feuille	After 1st interview	For work efficiency, layering instead of wrapping was chosen as the cooking method.
Mackerel simmered in tomato sauce	Mackerel with tomato sauce	After 1st interview	The mackerel, which tends to leave an odor, was oven-baked and served with a tomato sauce prepared separately.
Stewed pumpkin with minced meat	Stewed pumpkin with minced chicken thick sauce	After 1st interview	By using a minced meat paste, the amount of water released from the dish is reduced. Considering work efficiency, it was decided to pour the bean paste on top of the boiled pumpkin.
Apple	(Not served)	After 1st interview	Considering the deterioration of appearance due to discoloration, we stopped offering apples.
Meat and potato stew	Meat and potato stew	After 1st interview	Each ingredient was seasoned and cooked separately before serving.
Sandwich	Sandwich	After 2nd interview	The sandwiches were cut so that the cross section could be seen, and each sandwich was wrapped in plastic wrap.
Boiled egg	Boiled egg (with shell)	After 2nd interview	For hygienic reasons, the eggs were not cut in half, but served in the shell and uncracked.
Chilled tofu	Chilled tofu (Tofu was heated)	After 2nd interview	From a hygienic standpoint, the tofu was cooked.
Sushi wrapped in fried tofu	Sushi wrapped in fried tofu (Pickled ginger was served on the side)	After 2nd interview	Since pickles were not provided in the model menu plan, we decided to offer pickled ginger as a separate item.
Carrots (boiled)	Carrots (boiled), Stir-fried julienne carrots, Grated carrots	After 2nd interview	In order to keep the menu interesting, we increased the variety of cooking methods.

^*1^ The first and second interviews were conducted on Aug 24 and Oct 16, 2025, respectively.

**Table 8:** Final suggested model menu for three daily meals over 7 days of the week.

Meal	Day 1	Day 2	Day 3	Day 4	Day 5	Day 6	Day 7
Breakfast	Brown rice Grilled cod Thick omelet Stewed pumpkin with minced chicken thick sauce Vegetables Orange Milk	Bread roll Scrambled eggs Bacon Potato salad Glazed carrots Vegetables Fruit juice Yogurt	Brown rice Seasoned seaweed Spanish mackerel (with grated white radish) Thick omelet Stir-fried hijiki seaweed Seasoned simmered soy pulp with vegetables Vegetables Banana	Bread roll Sauteed eggs and spinach Tandoori chicken Sauteed shrimp and onions Glazed carrots Vegetables Banana Drinking yogurt	Brown rice Tofu and mushrooms in thick sauce Stir-fried julienne burdock root Vegetables Yogurt	Bread roll Scrambled eggs Bacon Lotus root salad Vegetables Banana Yogurt	Bread roll Sauteed eggs and spinach Boiled shrimp Grated carrots Vegetables Fruit juice Processed cheese
Lunch	Mackerel salad with udon noodles Deep-fried eggplant Sauteed bell peppers Vegetable juice	Rice cooked with green soy beans White fish in thick sauce Lotus root salad Sauteed mushrooms Vegetables	Sandwich Steamed soybeans Macaroni salad Vegetables Orange Milk	Brown rice Sauteed salmon and vegetables Steamed soybeans Vegetables Vegetable juice	Tri-color rice bowl Fluffy potato Stir-fried julienne carrots Vegetables Fruit juice	Sandwich Boiled chicken thigh meat Boiled egg Stir-fried julienne burdock root Vegetables Fruit juice	Sushi wrapped in fried tofu Gari (sweet and sour pickled ginger) Grilled salmon Thick omelet Seasoned simmered soy pulp with vegetables Vegetables
Dinner	Rice cooked with salmon Pork and cabbage mille-feuille Boiled egg Potato salad Greens with tofu, white sesame and white miso Vegetables	Brown rice Meat and potato stew Chilled tofu Thick omelet Stir-fried julienne burdock root Vegetables Kiwi fruit	Brown rice Chop suey Chinese steamed pork dumplings Boiled egg Stir-fried julienne carrots	Rice cooked with bamboo shoots Steamed chicken salad Chilled tofu Stewed pumpkin with minced chicken thick sauce Potato salad Vegetables Pineapple	Pork salad with udon noodles	Brown rice Mackerel with tomato sauce Thick omelet Macaroni salad Vegetables Processed cheese	Brown rice Tofu hamburger steak Macaroni salad Sauteed mushrooms Vegetables

The final suggested model menu was developed by the authors based on the two interviews conducted in 2025 with Hotel B in Tokyo.

**Figure 2:**
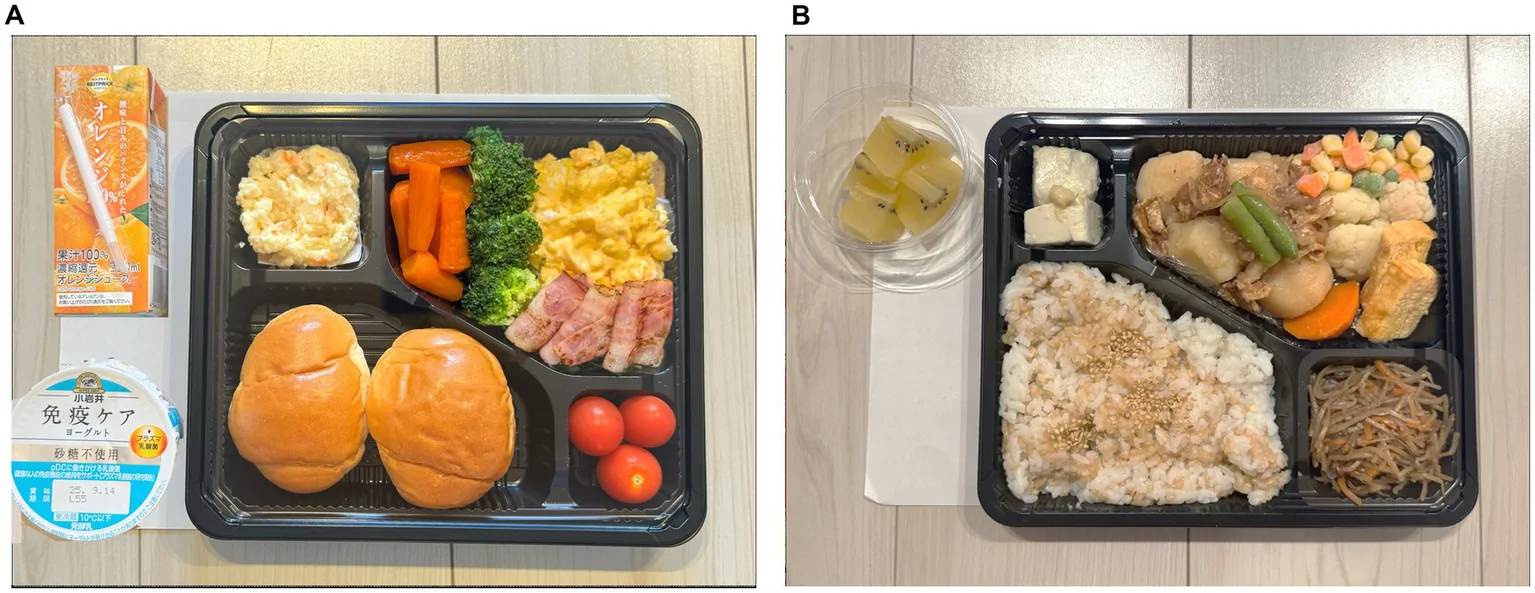
Examples of final suggested model menu. **(A)** Left: Day 2 breakfast: Bread roll with jam and butter, scrambled egg, baked bacon, potato salad, carrot glace, broccoli, tomatoes, orange juice, and yogurt with honey. **(B)** Right: Day 2 dinner: Brown and white-mixed-rice with sesame, Japanese meat and potato stew, rolled egg, burdock roots, cauliflowers, mixed vegetables, tofu, and kiwi.

#### 3.4.1 Increased vegetable servings

In the final suggested model menu, the average vegetable serving over the 7 days was 340 g. Daily vegetable serving on 4 days exceeded 350 g, and even on days when the serving was below 350 g, it was over 258.7 g.

Boiled/steamed was the most common cooking method (34.4%), followed by raw/salad (22.5%), soup (20.7%), and simmered (17.1%). Pickled and fried foods were served on only 1 day of the 7-day menu, while stir-fried food was served on 3 of the 7 days.

#### 3.4.2 Healthy cooking methods

Among the 21 model menu items, fried food was served once (fried eggplant). For main meat and fish dishes, such as “pork shabu-shabu,” “meat and potato stew,” and “tandoori chicken,” we intentionally reduced the amount of oil used by boiling, simmering, or grilling the dishes. In addition to reducing the number of rich, heavy dishes, mayonnaise and sesame oil were provided as optional seasoning to give flavor, with the amounts used adjustable by the individual.

The above two points resulted in the final suggested model menu that met the RVs for both vitamin C and salt, which were identified as problems in the dietary assessment of the Hotel B’s boxed meals, with an average of 186 mg and 6.6 g over 7 days, respectively.

### 3.5 Evaluation of the final suggested model menu in comparison with the “smart meal pattern”

We evaluated the final suggested model menu in comparison with the “Smart Meal Pattern” (Table 3). As a result, 57% of meals did not achieve the “appropriate range for protein–fat–carbohydrate (PFC) balance.”

## 4 Discussion

### 4.1 Dietary assessment of boxed meals served at hotel B during the fifth wave

Previous studies found that prepared foods and boxed meals from supermarkets and convenience stores typically contain excessive calories, fat, and salt, and lack vegetables (66–71). A similar tendency was observed in the Hotel B’s boxed meals.

The boxed meals provided by Hotel B to recovering patients were high in calories and sodium. Excessive salt intake is a cause of chronic diseases, such as hypertension, cardiovascular disease, and kidney disease. These underlying diseases have been reported as risk factors for the severe progression of pandemics, including COVID-19 (72–76).

Many studies show a relationship between vitamin C and immune function, such as increased consumption during colds and infections, because of the body’s increased inflammatory response (30, 77–79). We used mean vitamin C content of 7 days (78 mg/day) was used for assessment because day-to-day variability in vitamin C content assessed by coefficient of variation (0.23) was not as large as daily variation of vitamin C intake (0.85 for adult women and 0.98 for adult men) (80). Since the RV for vitamin C (80 mg/day) is the same value as Estimated Average Requirement for 15 years old and over (57), more than 50% of the patients could not fulfill their needs. Vitamin C is a nutrient mainly obtained from vegetables and fruits. Although vegetables were served every day, the average amount served was 234 g over the 7-day period, representing 66.8% of the target of 350 g or more established by Health Japan 21 (third term) (58).

Furthermore, fatty foods, including fried foods, take more difficult to digest and place greater burden on the gastrointestinal tract. On the days when four kinds of fried foods were served as “assorted fried food,” the fat energy ratio was 36%, exceeding the appropriate target range of 20–30% in the Dietary Reference Intakes for Japanese 2025 Edition (57). Therefore, the meals provided to recovering patients at hotel-based care facilities were considered inappropriate for their treatment from a nutritional standpoint.

These results can be attributed to concerns about safety and security in the hotel care system. The manual for hotels providing care for mild COVID-19 cases (5) published by the Ministry of Health, Labour and Welfare during the hotel care system provided several guidelines, stating:

The manual was created with the aim of creating a safe and secure care environment by providing specific instructions for the staff running the facility with regard to procedures and infection control measures, as well as specific precautions for patients with mild symptoms recuperating at the facility.

The importance of facility management and menu planning is the “safety and security of recuperating patients.” This was evident from concerns expressed about the uneven heating of individual menus (Table 7). There was also a need to carefully disinfect the areas used by patients after meal service (Table 2). In the past, there had been cases of food poisoning caused by inadequate hygiene management at disaster shelters, which were similar to the hotel environments providing care for COVID-19 patients. The Ministry of Agriculture, Forestry and Fisheries reminded people to be careful when cooking, such as not touching food with bare hands, avoiding raw foods, and providing dishes that had been thoroughly heated. The menu consisted of boiled, fried, and grilled foods (81, 82). Under the hotel-based system providing care for COVID-19 patients, the people consuming the food were considered “recuperating patients with reduced resistance;” hence, great care had to be taken to ensure the safety and security of food preparation and ingredients. Hotel B reported that even during normal hotel operations, meals are sometimes delivered to guestrooms as bento boxes (boxed meals), intended for immediate consumption after cooking and serving (Table 2). However, the boxed meals served during the COVID-19 pandemic were prepared as take-out meals, which required a certain amount of time between cooking and serving before eating. This required a more thorough control of temperature, based on Hazard Analysis and Critical Control Points (HACCP) (83), than in normal times, and cooking with oil became more common.

Thus, main dishes in the modified model menu were aimed to include tandoori chicken and tofu hamburger steak, which can be cooked with less oil than fried dishes. The spices used in tandoori chicken promote blood circulation, increase appetite, help with digestion, and function as antioxidants (84–86). Tofu, made from soybeans, and known as the “meat of the field” because of its high amino acid content, is more easily digested and absorbed than other soybean products. It is considered a highly effective food for patients recuperating from impaired gastrointestinal motility (87–89).

### 4.2 Evaluation of the final suggested model menu in comparison with the “smart meal pattern”

While the Smart Meal specifies the nutritional standards per meal, the final suggested model menu in this study was intended to achieve a daily nutritional balance. In addition, the RVs that we used for menu development, did not include fat-to-energy ratio and weight of vegetables, mushrooms, seaweed, and potato. Therefore, some of the final suggested model menu did not meet the recommended fat-to-energy ratio and weight of vegetables, mushrooms, seaweed, and potato of “Smart Meal” standard (Table 3).

#### 4.2.1 High fat-to-energy ratio

The main reason the fat-to-energy ratio exceeded the appropriate range was the use of seasonings. In response to comments that the nutritionally balanced boxed meals were “bland” (Table 2) (90, 91), we included separate seasoning sachets in the model menu so that the recovering patients could adjust the flavor to their taste. Since excessive salt intake is a risk factor for COVID-19, fats and oils, such as mayonnaise, sesame oil, and roasted sesame seeds, were mainly used. However, commercially available sachet seasonings have a fixed volume, and the final suggested model menu’s nutritional calculations were based on the contents of one sachet even if the patient did not use the whole sachet. Therefore, the fat-to-energy ratio was high.

#### 4.2.2 Short supply of vegetables

In a previous study of the nutritional characteristics of conventional ready-made boxed meals, the “short shelf-life of vegetables” and “differences in preservation characteristics” were cited as causes for the lack of vegetables in ready-made boxed meals (66). This suggested that including vegetables in ready-made bento boxes was difficult. Our first interview also revealed that “the storage space for foodstuffs was reduced by half due to zoning with recovering patients” (Table 2), that “it was necessary to heat the food in the boxed meal containers from the viewpoint of hygiene and safety compliance,” and that an operational system was required to “provide a maximum of 150 meals at 1,000 yen or less per meal.” These restrictions and conditions account for the absence of vegetables in the ready-made boxed meals mentioned above. It was difficult to provide “more than 140 g of vegetables per meal” in the final suggested model menu owing to the use of multiple foods with different preservation characteristics in the boxed meals. Meals that are time-consuming to prepare may pose a barrier to safely serving meals within a set timeframe with a limited number of employees. However, daily vegetable serving on 4 days exceeded 350 g that is a goal for vegetable consumption in Health Japan 21 (third term) (58), and even on days when the serving was below 350 g, it was over 258.7 g that was average intake in Japanese population (92).

### 4.3 What was learned from the interviews

#### 4.3.1 Importance of container (disposable bento box)

As reported from a disaster-affected facility (93), we also found that when preparing a 7-day menu, not all the dishes on the menu fit into the containers. On the contrary, there were some small gaps. Two types of containers were used in this study; in some cases, the meal fit well in one container but not in the other. The containers varied in size and in the number of compartments in the boxed meals used by Hotel B at the time. The interviews revealed that “the minimum lot size per type of container is fixed to some extent,” as “the space to store the ordered containers was limited under the restricted circumstances at that time.” Hence, “the standardization of containers contributed to the improvement of work efficiency and the prevention of errors.” Therefore, in emergencies, using multiple types of containers is difficult. In the case of meals served at a long-term care hospital affected by the 2024 Noto Peninsula Earthquake, the amount of food normally served could not be provided because the meal containers were too small; consequently, the residents lost weight and muscle mass due to a prolonged caloric deficit (93). Since meals at hotels providing care are also served in disposable boxed meal containers, the selection of containers also plays a key role in being able to provide meals that meet the minimum nutritional requirements. To enhance the feasibility of the final suggested model menu, it is necessary to confirm the availability and sizes of boxed meal containers used in this study at hotels and other accommodation facilities.

#### 4.3.2 Difficulty in serving soup

We planned to include hot soup in the model menu plan. Soup rehydrates the body, is easy to consume even when one is unwell, and facilitates the intake of all water-soluble nutrients present in the ingredients. Hot meals are also a factor affecting food satisfaction in stressful environments, such as disaster shelters and isolation areas (40). Thus, they are proposed as suitable during recuperation. Conversely, the interviews raised many concerns not only about the spread of infection and food poisoning but also about facility equipment, such as “pouring leftover liquids and ingredients down the drain,” the “risk of spreading infection by sharing cooking utensils,” and “difficulty in transporting liquids.” Providing soup during emergencies might pose a problem. Reports during the 2016 Kumamoto Earthquake indicated that some aged care facilities were unable to serve miso soup because it was difficult to transport due to elevator stoppages (94). In Hotel B, the elevator became unavailable due to the assignment of patients and staff who tested positive, necessitating the use of stairs to transport food and other items. Additionally, soups tend to have a higher salt equivalent than other side dishes and salads (67). Since the frequency of soup intake is associated with changes in salt intake (95), from the perspective of “serving at hotel care facilities,” serving soup has low priority.

### 4.4 How far nutritional adequacy should be pursued in emergency settings

Nutritional consideration is necessary in emergency settings in order to prevent secondary health damage by the crisis event. In refugee camps, undernutrition and acute malnutrition are common nutritional problems that lead to maternal and child death (96). However, among not hospitalized, mildly ill patients, weight loss along with poor appetite was assessed by dieticians as COVID-19-specific symptoms (97). In both cases, weight loss due to decrease in food intake should be addressed regardless of its cause; food insecurity or poor appetite (98). Plenty of fruits and vegetables and limited fried foods in the final suggested model menu could contribute to ease of eating among mildly ill patients.

Another important consideration in emergency settings is feasibility under limited resources; food, disposable container, staff, and equipment. The RVs were limited to energy and five nutrients considering feasibility from the past disaster experiences (55). In our menu development and modification, saving effort and infection prevention were regarded as equally important as nutritional adequacy.

### 4.5 Limitations

The present study has the following limitations.

The participants were from only one hotel. Patton mentioned “the logic and power of purposeful sampling lie in selecting information-rich cases for in-depth study” (99). Regarding this point, our target hotel was that from which we could learn a great deal about issues of central importance to the purpose of this study. In addition, the number of interviewees is limited to three. In the second interview, however, we asked if they had any additional opinions or suggestions on each day’s menu but only two comments were made (Table 2). We regarded this as data saturation.

The first author, the menu developer, attended each interview. Although the participants did not receive honoraria and not making money as individuals, there could be a possible risk of bias. Since she is an undergraduate student, however, they seemed easy to speak frankly.

The final suggested model menu that was considered feasible at Hotel B may be difficult to implement at other hotels. Feasible menu is different by restrictions that each hotel faced at that time, depending of their location, equipment, staffing, and budget etc. Although Hotel B was requested that standard meal cost be 1,000 yen per meal (including beverages) from the local government in advance, previous studies reported that many local governments had budgeted “2,500 to 3,000 yen per person per day,” and that some local governments outsourced or requested meals from known businesses or local restaurants (16). This qualitative study aimed to focus on one case and deeply explore very specific purpose, that is, a development of model menu that could be served under the identified restrictions on the operation of residential sanatoriums. Although our final suggested model menu may not be applicable for all the hotels in Japan but it could be served as a reference for those hotels that experienced similar restrictions.

## 5 Conclusion

During the COVID-19 pandemic, research and media reports indicated that patients housed in alternative accommodations were provided with nutritionally deficient meals, which impeded their convalescence. Hotel staff were not trained to meet the required nutritional standards. This study provided information on the matter by developing and accessing a model meal plan and service. The feasibility of the plan was assessed with key hotel staff. The study’s results highlight the importance of providing meals to vulnerable people during natural disasters or other emergencies, given the resource shortages, the reduced availability of facilities, and the psychological burden on those staying at the facilities and their caregivers.

## Data Availability

The datasets generated and/or analyzed during the current study are not publicly available because participants consented to the study on the condition that access to the data would be restricted to the research team. Therefore, the data cannot be shared with third parties.

## References

[1] World Health Organization. Coronavirus Disease (COVID-19). Available online at: https://www.who.int/health-topics (Accessed December 25, 2025).

[2] Ministry of Health, Labour and Welfare. (2023). Visualizing the Data: Information on COVID-19 Infections – Number of Confirmed Cases (Cumulative). Available online at: https://covid19.mhlw.go.jp (Accessed November 19, 2025).

[3] Ministry of Health, Labour and Welfare. (2023). Visualizing the Data: Information on COVID-19 Infections – Number of Deaths (Cumulative). Available online at: https://covid19.mhlw.go.jp (Accessed November 19, 2025).

[4] Ministry of Health, Labour and Welfare. (2023). Visualizing the data: Information on COVID-19 Infections – Trends in the Number of Severe Cases. Available online at: https://covid19.mhlw.go.jp (Accessed November 19, 2025).

[5] Ministry of Health, Labour and Welfare. (2020). Manual for Securing and Operating Hotels to Provide Lodging and Care for Patients With Mild COVID-19 Infection. 5th ed. (in Japanese). Available at: https://www.mhlw.go.jp/content/000740154.pdf (Accessed November 21, 2025).

[6] Ministry of Health, Labour and Welfare. (2020). Basic Approach to the Subjects of Medical Treatment for Patients With Mild Illness, etc. (in Japanese). Available online at: https://www.mhlw.go.jp/content/000618529.pdf (Accessed November 24, 2025).

[7] Calder PC. Nutrition and immunity: lessons from coronavirus disease-2019. Proc Nutr Soc. (2025) 84:8–23. doi: 10.1017/S0029665123004792, 37886807

[8] Briguglio M Pregliasco FE Lombardi G Perazzo P Banfi G. The malnutritional status of the host as a virulence factor for new coronavirus SARS-CoV-2. Front Med. (2020) 7:146. doi: 10.3389/fmed PMC7191079 32391367

[9] Cena H Chieppa M. Coronavirus disease (COVID-19–SARS-CoV-2) and nutrition: is infection in Italy suggesting a connection. Front Immunol. (2020) 11:944. doi: 10.3389/fimmu.2020.00944, 32574257 PMC7221157

[10] Shorrt KR Kedzierska K van de Sandt CE. Back to the future: lessons learned from the 1918 influenza pandemic. Front Cell Infect Microbiol. (2018) 8:343. doi: 10.3389/fcimb.2018.00343 PMC6187080 30349811

[11] World Health Organization Regional Office for the Eastern Mediterranean. Nutrition Advice for Adults during the COVID-19 Outbreak. Available online at: https://www.emro.who.int/nutrition (Accessed November 27, 2025).

[12] Sasagawa S. Honda Y. Morishita Y. (2022). What to do? Boxed Meals during COVID-19 Treatment: Considering Complaints and Criticisms. Asahi Shimbun. Available online at: https://www.asahi.com/articles (Accessed November 20, 2025).

[13] Tokyo Shimbun. (2022). Patients Recuperating at hotel care Facilities Served Famous Train Station Lunch Boxes In Response to Negative Reception, Gov. Koike Says “We’ll add a little color”. (in Japanese). Available online at: https://www.tokyo-np.co.jp/article (Accessed November 20, 2025).

[14] Sankei Shimbun. (2022). Osaka Prefectural Government Improves Boxed Meals at hotels Providing care. Available online at: https://www.sankei.com/article/20220809 (Accessed November 21, 2025).

[15] Mainichi Newspaper. (2021). Meals for COVID-19 Patients at hotels differ – Nutritional Management, lack of Consideration, Greasy and Lacking Vegetables. Available online at: https://mainichi.jp/articles/20210207/ddn/001/040/001000c (Accessed November 20, 2025).

[16] Takahashi H Sudo N. Meal provision for patients under hotel quarantine during the seventh wave of COVID-19. Jpn J Nutr Diet. (2025) 83:215–22. doi: 10.5264/eiyogakuzashi.83.215

[17] Japan Tourism Agency. (2025). Current Situation and Issues Surrounding Tourism. Available online at: https://www.mlit.go.jp/ (Accessed December 31, 2025).

[18] Ministry of Health, Labour and Welfare. (2025). Employment Trends Survey: Summary of Results. Available online at: https://www.mhlw.go.jp/toukei (Accessed December 31, 2025).

[19] Fujiyama M. Current Situation and Challenges of labor Shortage in the Tourism Industry. (2022). Tokyo: Japan Research Institute. Available online at: https://www.jri.co.jp/MediaLibrary/file/report/researchfocus/pdf/13866.pdf (Accessed December 25, 2025).

[20] Yamaguchi H. "On the human shortage and employment trends in the hotel industry – employment trends in the hotel industry and wholesale and retail trade, welfare services and other service industries. (in Japanese)". In: Proc 39th Natl Conf Japan Soc Tourism Res (2024). p. 57–62.

[21] Ministry of Health, Labour and Welfare. (1998). Act on Prevention of Infectious Diseases and Medical care for Patients with Infectious Diseases. Available online at: https://www.mhlw.go.jp/web/t_doc?dataId=79998826&dataType=0&pageNo=1 (Accessed December 25, 2025).

[22] Ministry of Health, Labour and Welfare. (1998). Ordinance for Enforcement of the Act on Prevention of Infectious Diseases and Medical Care for Patients With Infectious Diseases. Available online at: https://www.mhlw.go.jp/ (Accessed December 25, 2025).

[23] Central Disaster Management Council, Cabinet Office. (1961). Basic Plan for Disaster Prevention. Available online at: https://www.bousai.go.jp (Accessed December 25, 2025).

[24] Cabinet Office (Disaster Management). (2025). Utilization of Hotels and Inns and Efforts for Evacuation Shelters Other Than Designated Evacuation Shelters. Available online at: https://www.bousai.go.jp (Accessed December 25, 2025).

[25] Tsuboyama-Kasaoka N Hoshi Y Onodera K Mizuno S Sako K Factors impacting meal provision in emergency evacuation centers (shelters) after the great East Japan earthquake. J Japan Disaster Food Soc. (2014) 1:35–43.

[26] Tsuboyama-Kasaoka N Hoshi Y Onodera K Mizuno S Sako K. What factors were important for dietary improvement in emergency shelters after the great East Japan earthquake? Asia Pac J Clin Nutr. (2014) 23:159–66. doi: 10.6133/apjcn.2014.23.1.17, 24561984

[27] Tsuchida N Isobe S Watanabe S Ishigami K Yoshita K Yoshiike N . Changes in access to food and the frequency of food consumption before and after the Niigata Chuetsu earthquake: comparison between households in temporary housing and disaster-stricken housing. Jpn J Diet Assoc. (2010) 53:340–8.

[28] Kotani J Yamada I Ueda T. Kumamoto earthquake NST activity report: food problems in evacuation shelters in comparison with convalescent facilities. Ann Clin Nutr Metab. (2024) 16:173–80. doi: 10.15747/acnm.2024.16.3.173

[29] Yamaguchi J. How can we prevent malnutrition in times of disasters? J Nihon Univ Med Assoc. (2024) 83:231–5. doi: 10.4264/numa.83.6_231

[30] Ishigami A. Vitamin C deficiency and the importance of vitamin C intake in times of disaster. Vitamins (Kyoto). (2011) 85:400–4.

[31] Inagaki M. Exploration of Factors Behind Disaster-Related Deaths [Dissertation]. Joint Graduate School of Disaster Nursing. Hyogo: University of Hyogo (2019).

[32] Tsuboi M Sasaki H Park H Tsubokura M Sawano T Harada N . Medical perspective on the systemic challenges involving indirect disaster-related deaths in Japan. Sci Rep. (2025) 15:5225. doi: 10.1038/s41598-025-89349-7, 39939351 PMC11822013

[33] Cabinet Office (Disaster Management). (2024). Current Situation and Challenges of Evacuation Shelters. Available online at: https://www.bousai.go.jp (Accessed December 25, 2025).

[34] Haga Y Kim J Mitsuhashi N. The actual conditions and tendency of local facilities as specified refuge shelter in the Mid Niigata Prefecture Earthquake 2004 – a case study of refuge shelters in the former Nagaoka city. J Archit Plann (Trans AIJ). (2008) 73:349–55.

[35] Tham A. Hotel quarantine guest blogs: unpacking Scheiner's spaces of immobility and implications on urban tourism politics. Cities. (2023) 134. doi: 10.1016/j.cities.2023.104202

[36] Bastani P Samadbeik M. The experience of hotel quarantine during omicron variant of COVID-19. Arch Clin Infect Dis. (2022) 17. doi: 10.5812/archcid.123023

[37] Haire B Gilbert GL Williams JH Kaldor JM Hendrickx D Dawson A . Experiences of risk in Australian hotel quarantine: a qualitative study. BMC Public Health. (2022) 22. doi: 10.21203/rs.3.rs-879884/v1 PMC9097144 35549917

[38] Dincer D Gocer O. Quarantine hotels: the adaptation of hotels for quarantine use in Australia. Buildings. (2021) 11:617. doi: 10.3390/buildings11120617

[39] Chia KW Xiong J. Once upon a time in quarantine: exploring the memorable quarantine hotel experiences of Chinese student returnees during the COVID-19 pandemic. Tour Hosp Res. (2023) 23:72–87. doi: 10.1177/14673584221085209, 37520688 PMC9024064

[40] Hayakawa K Lee C Hosaka M Imura S Noguchi R Research on meal satisfaction in closed and isolated environments: review of disaster shelter meals based on meal satisfaction of Antarctic research expedition members. J Disaster Food Soc. (2024) 11:105–11.

[41] Shimizu M. Survey on accommodation facilities for new coronavirus infection control – actual conditions of accommodation facilities in the 5th wave. Nichi-Iryo Soken Res Rep. (2022):127

[42] Fanelli A Cescatti A Ciscar J Ciscar JC Dubois G Ibarreta D . Assessing the risk of diseases with epidemic and pandemic potential in a changing world. Sci Adv. (2025) 11. doi: 10.1126/sciadv.adw6363, 40700491 PMC12285694

[43] World Health Organization. The changing face of pandemic risk: how we need to adapt, protect and connect. Available online at: https://www.who.int/newsitem/05022025 (Accessed November 21, 2025).

[44] United Nations Department of Economic and Social Affairs, Population Division. World Population Prospects. (2024). Available online at: https://population.un.org (Accessed November 24, 2025).

[45] United Nations Department of Economic and Social Affairs, Population Division. (2025). World Urbanization Prospects 2025 Summary of Results. Available online at: https://www.un.org/development.pdf (Accessed November 24, 2025).

[46] Patton MQ. "Comparison-focused sampling options". In: Patton MQ, editor. The Qualitative Research and Evaluation Methods: Integrating Theory and Practice, fourth Edn. California: SAGE Publications, Inc. (2015). p. 277–82.

[47] Tokyo Metropolitan Government Task Force on New Coronavirus Infections. (2023). Tokyo Metropolitan Government’s Countermeasures Against New-Type Coronavirus Infections – Status From the First to the Sixth Wave. Available online at: https://www.bousai.metro.tokyo.lg.jp (Accessed November 26, 2025).

[48] Matsunaga N Akiyama T Asai Y Saito K Hayakawa K Suzuki S . COVTREGI-JP COVID-19 “5th Wave” Summary of the Registry Study on COVID-19. National Repository for Emerging and Re-Emerging Infections Data Bank Project. Available online at: https://icrown-portal.jihs.go.jp (Accessed November 26, 2025).

[49] Hijikata N Murakami K Uehara S Shibuya K Fukuda Y. Characteristics and courses of COVID-19 in patients from the first through fifth waves of the disease: focused on a single public health center in Tokyo, Japan. [Nihon koshu eisei zasshi] Japanese Journal of Public Health. (2024) 71:117–23. doi: 10.11236/jph.23-077, 38008459

[50] Tokyu Corporation. (2021). The 3rd Tokyu Group Environmental and Social Contribution Award. Available online at: https://www.tokyu.co.jp/company/information/pdf/k-info-20210715-1.pdf (Accessed November 25, 2025).

[51] Ministry of Education, Culture, Sports, Science and Technology. (2020). The Standard Tables of Food Composition in Japan, 2020 edition (8th Revision) eBook (except Chapter 2). Available online at: https://www.mext.go.jp/content/ (Accessed December 7, 2025).

[52] Hariya J Adachi M. Sonomanma obento card. 3rd ed. Tokyo: Gunyosha (2019).

[53] Shufu-no-Tomo Sha. 8th Revision quick Reference Index Food & Cooking Calorie Book Corresponding to the 8th Revised Table of Food Composition. Tokyo: Shufunotomo (2022).

[54] Ministry of Health, labour and Welfare. Nutritional Reference Values for Feeding at Evacuation Shelters for meal assessment within three months after the disaster. Available online at: https://www.mhlw.go.jp/content/000622115.pdf (Accessed May 26, 2026).

[55] Sudo N Shimada I Tsuboyama-Kasaoka N Sato K. Revising “nutritional reference values for feeding at evacuation shelters” according to nutrition assistance by public health dietitians based on past major natural disasters in Japan: a qualitative study. Int J Environ Res Public Health. (2021) 18:10063. doi: 10.3390/ijerph181910063, 34639364 PMC8508193

[56] Sato H Sudo N Takeda T Shimada I Tsuboyama-Kasaoka N. Revision of “nutritional reference values for feeding at evacuation shelters” and model menus: a qualitative study. J Am Nutr Assoc. (2024) 43:157–66. doi: 10.1080/27697061.2023.2241129, 37579054

[57] Ministry of Health, Labour and Welfare. (2025). Dietary Reference Intakes for Japanese (2025 Version): Report of the Formalization Study Committee. Available online at: https://www.mhlw.go.jp/s.html (Accessed February 3, 2026).

[58] Ministry of Health, Labour and Welfare. (2023). Explanatory Material for the Promotion of Health Japan 21 (Third term). Available online at: https://www.mhlw.go.jp (Accessed December 31, 2025).

[59] Britannica Japan Ltd. "Fried foods". In: Britannica International Encyclopedia, Small Encyclopedia. Tokyo: Britannica Japan K.K (2010)

[60] National Agriculture and Food Research Organization. Table 1. Approach to Classifying Food Preparation and Methods. Available online at: https://www.naro.go.jp (Accessed December 6, 2025).

[61] Ministry of Internal Affairs and Communications. (1990). Japan Standard Commodity Classification (Revised June 1990). Available online at: https://www.soumu.go.jp/toukei_toukatsu/index/seido/syouhin/2index.htm (Accessed December 7, 2025).

[62] Tsuda K Fushiki T Honda Y. "Chapter 2: theory and characteristics of cooking operation". In: Yamazaki H, editor. Culinary Science: Food Preparation and Dietary design. Tokyo: Nakayama Shoten (2018). p. 23–9.

[63] Ministry of Agriculture, Forestry and Fisheries. Overview of Ready-Made Meals and Eating Out (3): Market Size of Ready-Made Meals, Items Purchased, etc.). Available online at: https://www.maff.go.jp/j/budget/yosan_kansi/sikkou/tokutei_keihi/seika_R2/ippan/attach/pdf/R2_ippan-222.pdf (Accessed January 4, 2026).

[64] Smart Meal Certification System. What is Smart Meal? “Healthy Meal and Food Environment” Certification system. Available online at: https://smartmeal.jp (Accessed January 4, 2026).

[65] Ichikawa Y. The certification system for healthy meal and food environment, “smart meal.”. J Cookery Sci Jpn. (2019) 52:423–5.

[66] Chida M Tanaka K Sato M. Nutritional evaluation of bento boxes in Akita supermarkets from the perspective of smart meal standards. Jpn J Nutr Diet. (2023) 81:75–83.

[67] Hayashi F Sakaguchi K Abe C. Nutritional Characteristics of Boxed Lunches and Prepared Foods in Convenience Stores: Comparison with “Healthy diet” Standards. Report of a Joint Research Project Funded by the Ministry of Health, Labour and Welfare (Comprehensive Research Project on Measures to Prevent Lifestyle-Related Diseases, Including Cardiovascular Disease and Diabetes). (in Japanese). (2021). p. 47–69.

[68] Nishida M Yamamoto N. Research of nutrition value and food composition in the boxed meals sold at convenience stores. Bull Fac Educ Wakayama Univ. (2015) 65:151–6.

[69] Isobe E Murayama N. Characteristics of bentos sold in convenience stores according to nutrition facts label and food weight. Bull Soc Hum Life Stud. (2017) 8:1–14.

[70] Namba T Kushida O Murayama N. The relationship between amount of vegetables and energy, percentage energy from fat, and sodium chloride equivalent in the boxed meals sold in convenience stores. Niigata J Health Welfare. (2012) 12:28–34.

[71] Taniuchi N Nagao-Sato S Nishida I Akamatsu R. Characteristics of vegetables in commercial boxed lunches. Jpn J Nutr Diet. (2025) 83:140–50. doi: 10.5264/eiyogakuzashi.83.140

[72] Zhang X Chen B Jia P Han J. Locked on salt? Excessive consumption of high-sodium foods during COVID-19 presents an underappreciated public health risk: a review. Environ Chem Lett. (2021) 19:3583–95. doi: 10.1007/s10311-021-01257-0, 34093102 PMC8167309

[73] Iaccarino Idelson P Rendina D. Nutrition and the COVID-19 pandemic: three factors with high impact on community health. Nutr Metab Cardiovasc Dis. (2021) 31:756–61. doi: 10.1016/j.numecd.2020.12.001, 33549427 PMC7723026

[74] Min B Fairchild RL. Over-salting ruins the balance of the immune menu. J Clin Invest. (2015) 125:4002–4. doi: 10.1172/JCI84690, 26485281 PMC4639973

[75] Li X Alu A Wei Y Wei X Luo M. The modulatory effect of high salt on immune cells and related diseases. Cell Prolif. (2022) 55:e13250. doi: 10.1111/cpr.13250, 35747936 PMC9436908

[76] Ministry of Health, Labour and Welfare. (2021). Response to risk Factors for Severe Illness in COVID-19-Positive Patients. (in Japanese). Available online at: https://www.mhlw.go.jp/content/10900000/000823697.pdf (Accessed January 4, 2026).

[77] Fawzi W Bromage S Abioye AI. Effect of micronutrient supplements on influenza and other respiratory tract infections among adults: a systematic review and meta-analysis. BMJ Glob Health. (2021) 6. doi: 10.1136/bmjgh-2020-003176, 33472840 PMC7818810

[78] Hemilä H Chalker E. Vitamin C for preventing and treating the common cold. Cochrane Database Syst Rev. (2013):CD000980. doi: 10.1002/14651858.CD000980.pub3, 23440782 PMC8078152

[79] Kamohara S. Clinical evidence of functional ingredients for prophylaxis of COVID-19: update 2021. Funct Food Res. (2021) 17:75–104.

[80] Willet W. "Nature of variation in diet". In: Willet W, editor. Nutritional Epidemiology Third edition. Oxford: Oxford University Press (2013). p. 34–48.

[81] Ministry of Agriculture, Forestry and Fisheries. Food Poisoning Prevention Measures at Evacuation Shelters. (in Japanese). Available online at: https://www.maff.go.jp/j/syouan/saigai_syokutyuudoku.html (Accessed January 4, 2026).

[82] National Institute of Health Sciences. (2018). A Case of *Staphylococcus aureus* Food Poisoning Caused by Rice Balls in an Evacuation Shelter. (in Japanese). Available at: https://h-crisis.niph.go.jp/archives/103459/ (Accessed January 4, 2026).

[83] Ministry of Health, Labour and Welfare. (2017). Sanitation Management Guidelines for large-scale Food Preparation Facilities. (in Japanese). Available online at: https://www.mhlw.go.jp/file (Accessed December 18, 2025).

[84] Inada S. The Complete Guide to Spices to Help Master Their Unique Flavors. (in Japanese). Tokyo: Seitosha (2023).

[85] Indo Curry Girl. Helpful spice curry to Energize mind and body. (in Japanese). Tokyo: Ie no Hikari Kyokai (2020).

[86] Farrimond S. The Science of spice: Understand flavor Connections and Revolutionise your Cooking. Tokyo: Seibundo Shinkosha (2021).

[87] Matoba T Yoshida H Yonezawa D. Simplified nutritional evaluation of proteins of some soy products based on in vitro digestibility. J Jpn Soc Food Nutr. (1981) 34:415–21.

[88] Imaki M Miyoshi T Fujii M Takahashi H Nakamura T Tanada S. Study on digestibility and energy availability of daily intake food in Japanese. Part 1: kori-dofu and soybean. Jpn J Hyg. (1989) 44:714–8. 10.1265/jjh.44.714 2810871

[89] Machida Y. Edible soybean proteins and new uses. J Japan Oil Chem Soc. (1963) 12:461–7. doi: 10.5650/jos1956.12.461

[90] Akamatsu R Kushida O Takahashi N Kurotani K. Status of provision of smart meal and issues of continuation of the certification for the “healthy meal and food environment” among certified restaurants and ready-made meal companies: results of the survey targeting the first renewal companies. Jpn J Nutr Diet. (2021) 79:37–45.

[91] Chiba Y Ishii A Suzuki H Hayashi F. Sales of nutritionally balanced bento and examination of factors related to their selection: initiatives at local bento vendors. JJHEP. (2025) 33:110–22.

[92] Ministry of Health, Labour and Welfare (2024). Available online at: https://www.mhlw.go.jp/content/10900000/001603146.pdf (Accessed May 29, 2026).

[93] Kokura Y. Impact of the 2024 Noto peninsula earthquake on nutritional status in residents of an integrated medical and long-term care facility: a descriptive study. Nutrients. (2025) 17:506. doi: 10.3390/nu17030506, 39940364 PMC11820174

[94] Sato H Sudo N. Impact of utility damage on meals for older adults after the Kumamoto earthquake, Japan. Asian J Diet. (2024) 6:1–14.

[95] Tsuchihashi N Omori R. Examination of foods that affect salt intake. J Utsunomiya Jr Coll Hum Welfare Food Sci Nutr. (2020):31–3.

[96] Sphere Association. The Sphere Handbook. Geneva: Sphere Association (2018).

[97] Lawrence V Hickson M Weekes CR Weekes CE Julian A Frost G . A UK survey of nutritional care pathways for patients with COVID-19 prior to and post-hospital stay. J Hum Nutr Diet. (2021) 34:660–9. doi: 10.1111/jhn.12896, 33735530 PMC8250968

[98] Faradina A Tseng S-H Ho DKN Nurwanti E Hadi H Purnamasari SD . Adherence to COVID-19 nutritional guidelines is associated with better nutritional management behaviors of hospitalized COVID-19 patients. Nutrients. (2021) 13:1918 10.3390/nu13061918 PMC8226761 34204893

[99] Patton MQ. "Purposeful sampling and case selection: overview of strategies and options". In: Patton MQ, editor. The Qualitative Research and Evaluation Methods: Integrating Theory and Practice, fourth Edn. California: SAGE Publications, Inc. (2015). p. 264–72.

